# Development of a prognostic model for early-stage gastric cancer-related DNA methylation-driven genes and analysis of immune landscape

**DOI:** 10.3389/fmolb.2024.1455890

**Published:** 2024-10-30

**Authors:** Chen Su, Zeyang Lin, Zhijian Ye, Jing Liang, Rong Yu, Zheng Wan, Jingjing Hou

**Affiliations:** ^1^ The School of Clinical Medical, Fujian Medical University, Fuzhou, Fujian, China; ^2^ Department of Gastrointestinal Surgery, Zhongshan Hospital of Xiamen University, School of Medicine, Xiamen University, Xiamen, China; ^3^ Institute of Gastrointestinal Oncology, School of Medicine, Xiamen University, Xiamen, China; ^4^ Department of Pathology, Zhongshan Hospital of Xiamen University, School of Medicine, Xiamen University, Xiamen, China; ^5^ Department of Pathology, The Third Affiliated Hospital of Sun Yat-sen University, Guangzhou, China; ^6^ Department of Minimally Invasive and Interventional Therapy for Cancer, Zhongshan Hospital of Xiamen University, School of Medicine, Xiamen, China

**Keywords:** gastric cancer, FAAH, C1orf35, DNA methylation-driven gene, prognosis

## Abstract

**Background and Aims:**

This study aimed to develop a prognostic model based on DNA methylation-driven genes for patients with early-stage gastric cancer and to examine immune infiltration and function across varying risk levels.

**Methods:**

We analyzed data from stage I/II gastric cancer patients in The Cancer Genome Atlas which included clinical details, mRNA expression profiles, and level 3 DNA methylation array data. Using the empirical Bayes method of the limma package, we identified differentially expressed genes (DEGs), and the MethylMix package facilitated the identification of DNA methylation-driven genes (DMGs). Univariate Cox regression and LASSO (least absolute shrinkage and selector operation) analyses were utilized to pinpoint critical genes. A risk score prediction model was formulated using two genes that demonstrated the most significant hazard ratios (HRs). Model performance was evaluated within the initial cohort and verified in the GSE84437 cohort; a nomogram was also constructed based on these genes. We further examined 50 methylation sites associated with three CpG islands in C1orf35 and 14 methylation sites linked to one CpG island in FAAH. The CIBERSORT package was employed to identify immune cell clusters in the prediction model.

**Results:**

A total of 176 DNA methylation-driven genes were refined down to a four-gene signature (ZC3H12A was hypermethylated; GATA3, C1orf35, and FAAH were hypomethylated), which exhibited a significant correlation with overall survival (OS), as evidenced by *p*-values below 0.05 following univariate Cox regression and LASSO analysis. Specifically, for the risk score prediction model, C1orf35, which had the highest hazard ratio (HR = 2.035, *p* = 0.028), and FAAH, with the lowest hazard ratio (HR = 0.656, *p* = 0.012), were selected. The Kaplan–Meier analysis demonstrated distinct survival outcomes between the high-risk and low-risk score groups. The model’s predictive accuracy was confirmed with an area under the curve (AUC) of 0.611 for 3-year survival and 0.564 for 5-year survival. Notably, the hypomethylation of the three CpG islands in C1orf35 and the single CpG island in FAAH was significantly different in stage I/II gastric cancer patients compared to normal tissues. Additionally, the high-risk score group showed a notable association with resting CD4 memory T cells.

**Conclusion:**

Promoter hypomethylation of C1orf35 and FAAH in early-stage gastric cancer underscores their potential as biomarkers for accurate diagnosis and treatment. The developed predictive model employing genes affected by DNA methylation serves as a crucial independent prognostic factor in early-stage gastric cancer.

## Introduction

Gastric cancer is globally recognized as the sixth most prevalent type of cancer and ranks as the seventh leading cause of cancer-related mortality, as reported by the World Health Organization (WHO) (https://gco.iarc.who.int/Data version: Globocan 2022 (version 1.1) 08.02.2024). The primary treatment strategy for this malignancy is surgery. The survival rates of patients undergoing potentially curative surgery vary significantly, influenced by factors such as the stage of the cancer at diagnosis and the quality of the surgical procedure (Huang et al., 2022; Orman and Cayci, 2019). Despite achieving successful R0 resections, which indicate no residual microscopic disease, some patients may experience recurrence due to previously undetected micro-metastases. To address this, gastrectomy followed by adjuvant chemotherapy is frequently utilized to diminish the risk of recurrence. Prior research highlights the significant role of adjuvant chemotherapy in enhancing outcomes for patients with treatable advanced gastric cancer (Sakuramoto et al., 2007; Nakajima et al., 2010). While many patients with stage I or II gastric cancer experience positive outcomes following either endoscopic or traditional surgical interventions, the prognosis for others remains poor (Miyahara et al., 2022). Recent studies have focused on identifying clinicopathological factors associated with overall survival (OS) in the early stages of gastric cancer (Ikoma et al., 2016; Bausys et al., 2018; Aoyama et al., 2014; Saito et al., 2016), yet the genetic markers predictive of prognosis at these stages are still not well-defined. The identification of such predictive markers is essential for improving survival forecasts for patients with early-stage gastric cancer.

Alterations in DNA methylation, including increased methylation of tumor-suppressor genes (Manel, 2002) and decreased methylation of oncogenes (Feinberg and Vogelstein, 1983), play a pivotal role in the pathogenesis of various cancers, including gastric cancer (Tahara and Arisawa, 2015; Calcagno et al., 2013). Genes such as SFRP2, THBS1, and UCHL1, which exhibit aberrant methylation patterns, could be crucial in determining the prognosis of gastric cancer (Yan et al., 2021; Hu et al., 2021; Wang et al., 2015). The tumor microenvironment (TME) is a complex network, and earlier research has suggested that tumor-infiltrating immune cells (TIICs) significantly influence the initiation, progression, and clinical outcomes of cancer. Additionally, the responses of innate and adaptive immune systems are critical determinants of the efficacy of immunotherapies (Steidl et al., 2010; Guo et al., 2021; Seager et al., 2017). Recent studies have elucidated the interaction between DNA methylation and tumor immunity, revealing that DNA methylation regulation-related genes (DMRegs) have potential effects on immune cell infiltration, the TME, and the efficacy of immunotherapy in hepatocellular carcinoma (HCC) patients. High scores in DMRegs, characterized by the predominance of TP53 wild-type mutations, elevated expression of PD-1 and CTLA-4, and marked immune activation, correlate with a poor prognosis (Song et al., 2022). Another study also confirmed the significant role of DNA methylation in influencing tumor immunity, although its comprehensive impact on TME formation and immune activation remains to be fully elucidated (Yuan et al., 2022; Suarez-Alvarez et al., 2012). These findings underscore the close relationship between DNA methylation and immune regulation, meriting further investigation. However, the specific effects of DNA methylation on the prognosis of early gastric cancer and the functionality of TIICs remain ambiguous. Consequently, further research into DNA methylation could offer valuable insights into this field.

This study aims to identify genes regulated by DNA methylation and explore the relationship between these DMGs and TIICs, which may serve as prognostic indicators for patients with stage I or II gastric cancer. This research could significantly enhance our understanding of the characteristics of tumor microenvironment cell infiltration and inform treatment strategies. Our findings suggest that methylation modifications of several genes are intricately linked to the early development of gastric cancer.

## Material and methods

### TCGA DNA methylation and gene expression data

We procured level 3 DNA methylation and mRNA expression datasets, along with corresponding clinical data for stage I/II gastric cancer, from TCGA (Atlas, 2014). This included mRNA expression data for 164 tumor and 32 normal tissues and DNA methylation data for 189 tumor and 27 normal tissues (Zhu et al., 2014). Clinical details such as age, gender, and stage were also collected (Table 1). Both the methylation and mRNA expression data were generated using the Illumina Infinium Human Methylation450 BeadChip and Illumina GA_RNASeq V2.1.0.0 platforms, respectively (Illumina, Inc., San Diego, CA, United States).

**TABLE 1:** Clinical characteristics of the study population.

Clinicopathological parameter	Training cohort (TCGA)	Validation cohort (GSE84437)	*P*-value
Age, years			
<65	77	93	<0.001
≥65	109	57	
Sex			
Male	121	100	0.612
Female	68	50	
T stage			
T1, T2	91	40	<0.001
T3, T4	98	110	
N stage			
N0	125	79	0.005
N+	60	71	

### Identification of DEGs between GC and normal tissues

Employing the limma package in R (Smyth et al., 2005), we identified DEGs between tumor and normal gastric tissues. Expression fold-change (FC) was calculated, and DEGs were selected based on a significance threshold of *p* < 0.05 and |log2FC| ≥ 0.585.

### Comprehensive analysis of DNA methylation and gene expression

To identify differentially hyper- and hypomethylated genes, we used the MethylMix package in R (Gevaert, 2015; Cedoz et al., 2018). DMGs were classified as those if they satisfied the following criteria: *p* < 0.05, |log2FC| ≥ 1 and cor ≤ −0.3. The MethylMix analysis was conducted in three stages: initially, we overlaid cancer DNA methylation data with corresponding data of gene expression for pinpointing methylation changes impacting gene expression. For additional examination, we only chose those genes satisfying the criteria for correlation filtering. Next, we implemented a beta mixed model to describe the methylation patterns across an extensive cohort of patients, reducing requirements for a preset threshold. Finally, we employed the Wilcoxon rank-sum test for contrasting the DNA methylation levels between gastric cancer and normal tissues.

### Univariate Cox regression analysis

Utilizing the Survival package in R, we carried out a univariate Cox regression analysis. This analysis focused on DMGs correlated with patient outcomes, calculating hazard ratios and their confidence intervals. We set the significance at a *p*-value less than 0.05.

### Identifying critical DNA methylation-driven genes via LASSO

We utilized LASSO analysis to explore how the expression of genes influenced by DNA methylation correlates with prognosis. This method effectively identified key genes driven by DNA methylation that are significantly linked to prognosis, enhancing the model’s accuracy and minimizing the likelihood of overfitting.

### Development and validation of the risk score model

From the univariate Cox regression and LASSO analyses, we selected the two methylation-driven genes with the highest or lowest hazard ratios to develop a risk score prediction model. This model utilized a linear combination of gene expression levels, each weighted by coefficients derived from multivariate Cox regression analysis. Employing this model, we split gastric cancer patients into categories of high and low risk in accordance with an optimal risk score threshold. We obtained the risk score for each patient by Risk score = Expression methylation-driven gene 1 × Coefficient methylation-driven gene 1 + Expression methylation-driven gene 2 × Coefficient methylation-driven gene 2. We assessed survival differences between the high-risk and low-risk groups using Kaplan–Meier survival plots. The GSE84437 dataset from the GEO database (https://www.ncbi.nlm.nih.gov/geo/) served to validate the prognostic model. Additionally, for evaluating the model’s predictive performance, we conducted a time-dependent ROC analysis.

### Development and assessment of the nomogram in the TCGA dataset

We built a nomogram for predicting the 1-, 3-, and 5-year survival outcomes for stage I/II GC patients using two methylation-driven genes. The calibration and discrimination of the nomogram were evaluated through a bootstrap approach involving 1,000 resamples.

### Clinical samples

The research methodologies were sanctioned by the Institutional Medical Ethics Committee at Xiamen University. We collected clinical samples after obtaining informed consent from the patients, in compliance with the Declaration of Helsinki (1975) guidelines. The diagnosis of GC was validated by two expert pathologists. From Zhongshan Hospital of Xiamen University, 36 stage I and II human gastric cancer specimens, along with adjacent epithelial tissues, were collected. Inclusion criteria were as follows: (I) a pathological diagnosis of GC; (II) undergoing radical surgery; (III) classified as stage I/II according to the TNM Classification of Malignant Tumors, 8th edition; and (IV) availability of complete postoperative information. Exclusion criteria included: (I) patients under 18 years of age; (II) missing clinicopathological or follow-up information; and (III) a background of other or additional primary malignancies.

### DNA methylation detection

Following the protocol provided by the manufacturer, genomic DNA was extracted from paraffin-embedded tumor and control tissue samples from stage I and II patients using the QIAGEN kit, Hilden, Germany. The extracted DNA was then measured and adjusted to a working concentration of 20 ng/μL. Selection of CpG islands in the proximal promoter regions of the C1orf35 and FAAH genes adhered to specific criteria: (1) they must be at least 200 base pairs in length; (2) they should have a GC content of at least 50%; and (3) they need an observed/expected CpG dinucleotide ratio of 0.60 or above. A total of 50 CpG methylation sites across three islands of the C1orf35 gene and 14 CpG methylation sites across one island of the FAAH gene were sequenced.

For the purpose of bisulfite conversion, 400 ng of genomic DNA underwent treatment with the EZ DNA Methylation™-GOLD Kit from ZYMO RESEARCH, located in CA, United States. Samples that did not achieve a bisulfite conversion rate of at least 98% were not included in the study. PCR products designed to target specific CpG sites were subjected to separation through agarose gel electrophoresis. Next, we employed the QIAquick Gel Extraction Kit from QIAGEN in Hilden, Germany, to purify these products. Methylation analysis was then performed using the Illumina HiSeq/MiSeq 2000 systems according to the guidelines provided by the manufacturer. For further details on the CpG sites analyzed, refer to Table 2.

**TABLE 2:** Clinical characteristics of the study population.

Target	Chr	Start	End	Length	PrimerF	PrimerR
C1orf35-1_1	chr1	228103687	228103869	183	TTATTTAGGAGGTTGAAGTAGGAGAA	TCCCRCCTCAACCTCAAAAC
C1orf35-2_1	chr1	228102175	228102341	167	AAGYGAGTTTTGYGGAGGAGTTT	AAACCRCCAAACTAAAACTATCTATATTCAC
C1orf35-3_1	chr1	228103237	228103418	182	AAGGTAGTTGTTTGGGGTTTG	CRAAAACACAAATCCRAAACAATAC
FAAH-1_2	chr1	46394679	46394483	197	GGGTAAGGAGAGATTTTGGAGAGTT	ACAAAAACAACRAACRAACCTAAAAA

### Analysis of immune cell infiltration in the tumor microenvironment (TME)

We used the CIBERSORT package to assess the distribution of 22 immune cell types from high-risk and low-risk groups respectively, enabling us to calculate the enrichment scores for every immune-related phrase in each group (Newman et al., 2015). First, we quantified and evaluated the relative abundance of different immune cell types between tumor and normal samples in the high-risk and low-risk groups to compare and predict immune cell infiltration between the two groups respectively; second, the distribution of immune cells between high-risk and low-risk groups was evaluated too. The Pearson correlation coefficient was used to test whether the relationship between tumor and normal samples is significant in two groups respectively (The threshold for significance: *p* < 0.05, | cor | ≥ 0.2).

### Gene Ontology enrichment analysis and GSEA

Venn diagram was employed to identify the key genes only high expressed in the high-risk group. The “clusterProfiler” R package was used to perform Gene Ontology enrichment analyses to elucidate whether the biological functions in genes are only associated with the high-risk group (Lu et al., 2008).

We performed GSEA to clarify the biological roles of previously identified methylated regulatory genes. The threshold for significance was established at *p* < 0.05, and a minimum enriched gene count of 15 was required.

### Statistical analysis

Analyses were conducted with IBM SPSS Statistics 27, utilizing chi-square tests, and outcomes reached statistical significance when the *p*-value was below 0.05.

## Results

### DEGs between tumor and normal groups

**FIGURE 1:**
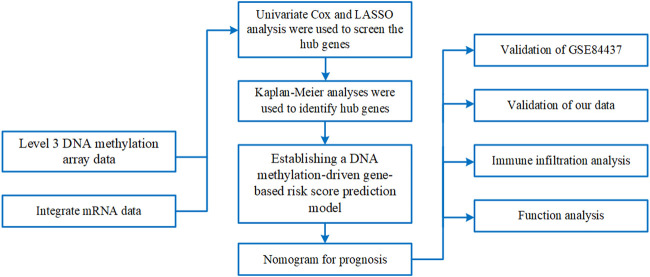
Methodological flowchart of the investigation.

A methodological flowchart is presented in Figure 1. We analyzed mRNA expression data from the TCGA database and identified 5,304 DEGs, with 4,732 being upregulated and 572 downregulated.

### Identification of DNA methylation-driven genes in gastric cancer

We identified 176 genes influenced by DNA methylation, comprising 38 hypermethylated and 138 hypomethylated genes. These genes demonstrated a correlation coefficient below −0.3 with DEGs and significant associations (adjusted *p*-value <0.05), prompting further detailed analyses.

### Connection between prognosis and genes affected by DNA methylation

**FIGURE 2:**
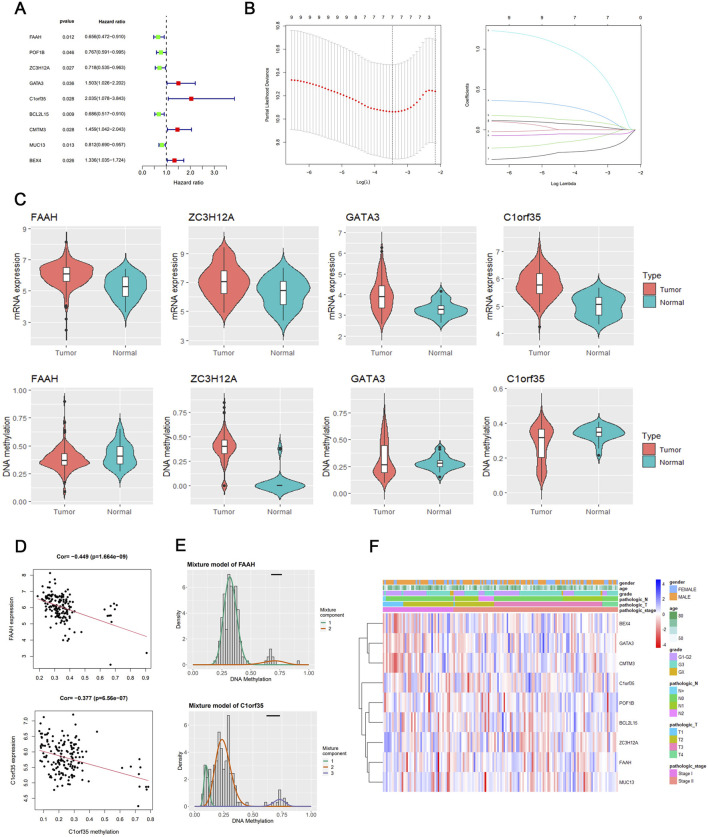
Texture feature selection and two-gene risk score model construction in the TCGA cohort. **(A)** Results of univariate analyses of DNA methylation-driven genes. **(B)** Identification of hallmark genes using LASSO regression. **(C)** mRNA expression and DNA methylation levels of the four DNA methylation-driven genes. **(D)** Regression analysis of the relationship between mRNA and DNA methylation levels of FAAH and C1orf35. **(E)** Differential methylation statuses of FAAH and C1orf35, depicted through histograms highlighting the distribution of methylation in gastric cancer (GC) samples. Beta values indicate the methylation level, ranging from 0 to 1, with the horizontal black bar representing the distribution of methylation values in non-tumorous gastric samples. **(F)** Heatmap of DNA methylation-driven genes.

A univariate Cox regression analysis was conducted to assess the prognostic impact of these methylation-altered genes. Genes such as GATA3, C1orf35, CMTM3, and BEX4, which exhibited hazard ratios greater than 1, were deemed independent risk factors. Conversely, genes including FAAH, POF1B, ZC3H12A, BCL2L15, and MUC13, with hazard ratios less than 1, were considered protective (Figure 2A). A heatmap of these DNA methylation-driven genes is shown in Figure 2F.

Identifying critical genes using LASSO analyses and building a risk score prediction model based on genes influenced by DNA methylation.

**FIGURE 3:**
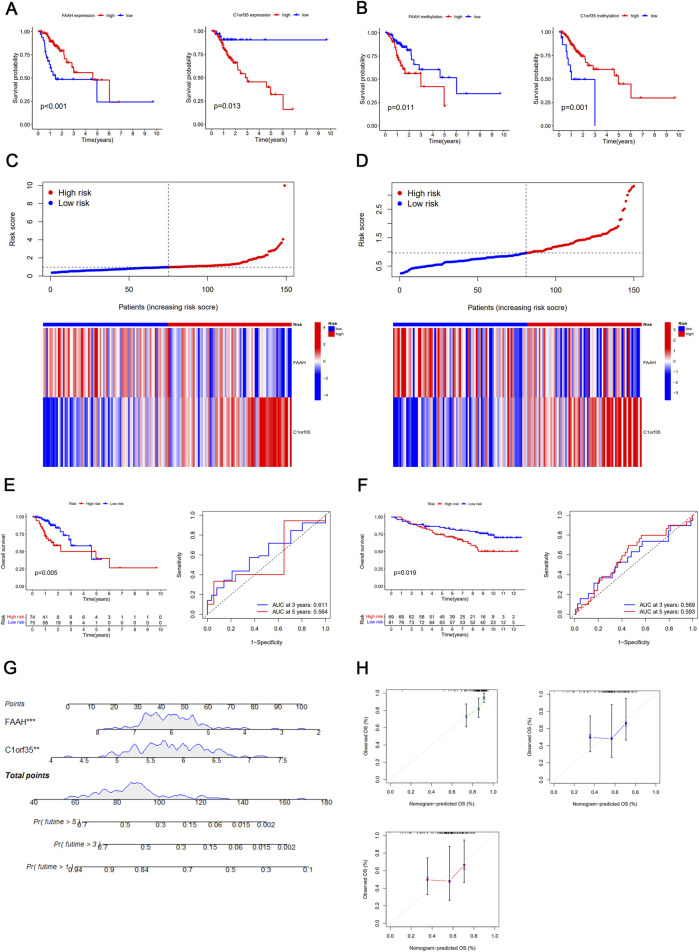
**(A)** Kaplan–Meier survival curve of DNA methylation-driven gene FAAH and C1orf35. **(C)** Heatmap and distribution of the two gene expression profiles in the high-risk and low-risk subgroups in the TCGA database. **(D)** Heatmap and distribution of the two gene expression profiles in the high-risk and low-risk subgroups in the GEO database. **(E)** Kaplan–Meier survival curve of DNA methylation-driven gene-based risk score prediction model and time-dependent ROC in the TCGA database. **(F)** Kaplan–Meier survival curve of DNA methylation-driven gene-based risk score prediction model and time-dependent ROC in the GEO database. **(G)** Nomogram for predicting the probability of 1-, 3-, and 5-year survival times for patients with stage I/II GC. **(H)** Calibration curve for the risk score model in the validation cohort. The dotted line represents the ideal predictive model, and the solid line represents the observed model.

The nine selected DNA methylation-driven genes underwent 1,000 iterations of LASSO regression to further narrow the selection. Four genes were identified using LASSO. Cross-validation determined the optimal adjustment parameter λ, minimizing the error rate (Figure 2B). The levels of expression and methylation of these genes are displayed in Figure 2C. Based on their respective hazard ratios—with C1orf35 having the highest (HR = 2.035, *P* = 0.028) and FAAH the lowest (HR = 0.656, *P* = 0.012)—and significant Kaplan–Meier survival analyses (*P* < 0.05), these two genes were selected to construct a risk score prediction model based on DNA methylation-driven gene activity. The expression of FAAH and C1orf35 have negative correlaion with methylation of these two genes (Figure 2D). The Mixture model of FAAH and C1orf35 have been shown in Figure 2E. Subsequent survival analysis and ROC curve assessments were performed on this model (Kamarudin et al., 2017). The Kaplan–Meier plots revealed a significantly shorter OS for the group with elevated risk scores (*P* = 0.005) (Figure 3E), with AUC values of 0.564 for 5-year survival and 0.611 for 3-year survival, respectively (Figure 3E).

### Risk score model validation

The effectiveness of the predictive signature was evaluated using the TCGA dataset and further confirmed through the GSE84437 dataset. In the high-risk group, OS was notably reduced (*P* = 0.019), as indicated by the Kaplan–Meier analysis (Figure 3F). The AUC values were recorded at 0.569 for 3-year and 0.593 for 5-year survival periods, respectively (Figure 3F).

### External validation of the nomogram in the TCGA cohort

Our nomogram, incorporating a DNA methylation-based gene signature, demonstrated broad applicability for both long- and short-term patient follow-ups, as depicted in Figure 3G. The calibration curves closely matched the predicted probabilities of OS at 1-, 3-, and 5-year intervals for gastric cancer patients (Figure 3H).

### Methylation level of C1orf35 and FAAH in the early-stage gastric cancer patients

**FIGURE 4:**
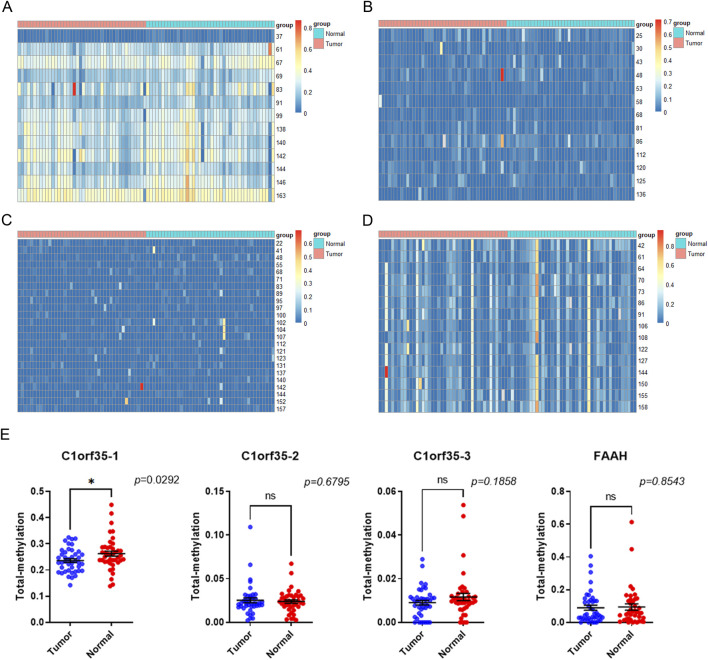
Heatmap target T vs. N. no cluster. **(A) (B) (C)** The heatmap shows the differential methylation sites on the three CPG islands of the C1orf35 gene and **(D)** methylation sites on the 1 FAAH CpG islands in stage I/II GC patients (T: tumor; N: Normal). **(E)** C1orf35 and FAAH island methylation level in stage I/II GC patients.

To assess the methylation levels in early-stage gastric cancer, tissue samples from 34 patient pairs were examined. We calculated the methylation level at each CpG site as the proportion of methylated cytosine to the total cytosines examined. A total of 50 methylation sites associated with three CpG islands in C1orf35 and 14 methylation sites associated with one CpG island in FAAH were analyzed (Figures 4A–D). The heatmap indicates variance in methylation levels across samples. Notably, methylation was lower in one island of C1orf35 (C1orf35_1: *p* = 0.0292; and not statistical significance in the single island of FAAH (*p* = 0.8543) compared to normal tissues, as shown in Figure 4E.

### Analysis of immune cell infiltration in the tumor microenvironment (TME)

**FIGURE 5:**
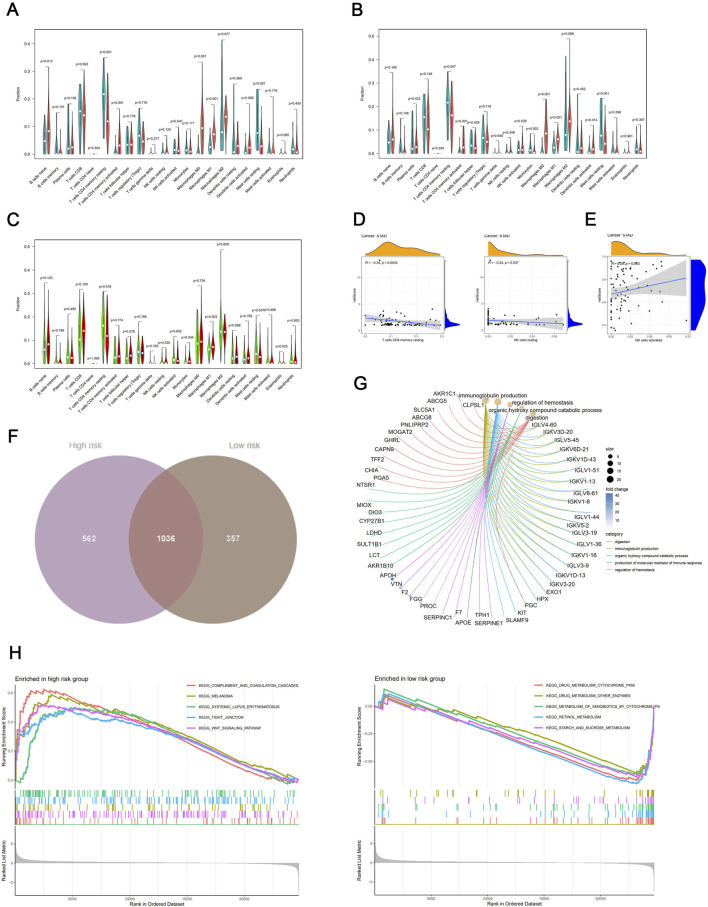
**(A)** Differential expression of immune cell sets by ssGSEA between tumor and normal samples in the high-risk score group. Rose represents tumor, and cyan represents normal. **(B)** Differential expression of immune cell sets by ssGSEA between tumor and normal samples in the low-risk score group. Rose represents tumor, and cyan represents normal. **(C)** Differential expression of immune cell sets by ssGSEA between high-risk score and low-risk score groups. Red represents the high-risk score group, and green represents the low-risk score group. **(D)** Positive correlation between ssGSEA scores of immune cells and risk score in the high-risk score group. **(E)** Positive correlation between ssGSEA scores of immune cells and risk score in the low-risk score group. **(F)** Venn diagram illustrating the overlap of immune cells between high-risk score and low-risk score groups. **(G)** Cnetplot depicting the network of marker genes from these pathways in the high-risk score group. Colored points indicate corresponding pathways. **(H)** Enrichment plots of the top five KEGG pathways in the high-risk score and low-risk score groups for stage I/II GC.

First, we quantified and evaluated the relative abundance of different immune cell types between tumor and normal samples in the high-risk and low-risk groups. In the high-risk group, B cells naive, CD4 memory resting, CD4 memory activated, macrophages M0, dendritic cells activated, and mast cells resting have statistically significant differences (Figure 5A), and CD4 memory resting, CD4 memory activated, monocytes, macrophages M0, macrophages M1, dendritic cells activated, and mast cells resting cells also have statistically significant differences (Figure 5B). Second, the distribution of CD4 memory resting, Monocytes, Macrophages M1, Mast cells resting and Eosinophils cells between high-risk and low-risk groups have a statistically significant difference (Figure 5C). The Pearson correlation analysis showed that T cells CD4 memory resting and NK cells resting are associated with the risk score in the high-risk group (Figure 5D), and NK cells activated is positive with risk score in the low-risk group (Figure 5E). Finally, only NK cells resting were observed to be positive in both scores of immune cell infiltration and correlation analysis.

### Functional annotations

We obtained the key genes only highly expressed in the high-risk group for further analysis (Figure 5F). Figure 5G displays the Gene Ontology (GO) analysis results, which show that the marker genes of the B cells from these pathways, such as digestion, immunoglobulin production, organic hydroxy compound catabolic process, production of molecular mediator of immune response, and regulation of hemostasis.

We employed gene set enrichment analysis (GSEA) for identifying key signaling pathways associated with the risk score model in both groups. Pathways with a false discovery rate (FDR) below 0.05 and an enrichment score (ES) above 0.5 were considered significant. In the high-risk group, the most enriched pathways included “COMPLEMENT AND COAGULATION CASCADES,” “MELANOMA,” “SYSTEMIC LUPUS ERYTHEMATOSUS,” “TIGHT JUNCTION,” and “WNT SIGNALING PATHWAY” (risk score <0.314). Conversely, the low-risk group showed enrichment in pathways of “STARCH AND SUCROSE METABOLISM,” “RETINOL METABOLISM,” “METABOLISM OF XENOBIOTICS BY CYTOCHROME P450,” “DRUG METABOLISM OTHER ENZYMES,” and “DRUG METABOLISM CYTOCHROME P450” (Figure 5H). These findings underscore the potential molecular mechanisms driving tumor progression in GC.

## Discussion

In recent years, a significant body of research has focused on identifying clinicopathological factors associated with overall survival in stage I/II gastric cancer. However, only few studies have investigated genetic prognostic markers (Ikoma et al., 2016; Bausys et al., 2018; Aoyama et al., 2014; Saito et al., 2016). Our study aimed to develop a risk prediction model based on genes affected by DNA methylation in patients with stage I/II gastric cancer. This model seeks to identify patients who may benefit from more aggressive treatment strategies, including adjuvant chemotherapy.

Our initial analyses integrated microarray studies and bioinformatics techniques to identify DNA DMGs in stage I/II gastric cancer. Notable genes identified with differential methylation included ZC3H12A, GATA3, C1orf35, and FAAH. ZC3H12A, also known as MCPIP1, is an RNAse that acts as a novel suppressor of microRNA activity and biogenesis (Tahara et al., 2013). GATA3, a zinc-finger pioneer transcription factor, plays critical roles in gene regulation by binding to nucleosomal DNA and facilitating chromatin remodeling (Qiang et al., 2023). The chromosome 1 open reading frame 35 (C1orf35) gene (Melton et al., 2019) and fatty acid amide hydrolase (FAAH), responsible for the degradation of anandamide into arachidonic acid and ethanolamine, are also implicated in significant cellular pathways (Melton et al., 2019). Abnormal methylation of these genes can lead to various diseases, including cancer. Therefore, we conducted a thorough analysis and clinical validation of FAAH and C1orf35 as key components of our prognostic model.

Our risk score prediction model, based on univariate Cox regression and LASSO analyses, identified FAAH and C1orf35 as critical genes. Methylation sequencing experiments demonstrated significant hypomethylation in the promoters of these genes among the patients studied. This methylation pattern was confirmed in 34 pairs of early-stage gastric cancer patients. The Kaplan–Meier analysis validated the effectiveness of this prognostic model in predicting outcomes for stage I/II gastric cancer patients, and time-dependent ROC analysis further confirmed the model’s prognostic relevance.

A notable observation from our study was the prognostic significance associated with the methylation status of C1orf35 and FAAH. C1orf35, identified in multiple myeloma cell lines, acts as an oncogene promoting the G1-to-S cell cycle transition by modulating c-MYC expression. Its oncogenic activity may be inhibited by targeting c-MYC (Luo et al., 2020). Further studies have suggested a potential role for C1orf35 in liver cancer (Meier et al., 2021). Consistent with these findings, upregulated and hypomethylated C1orf35 was associated with poor prognosis in stage I/II gastric cancer patients.

FAAH, associated with cellular membranes, facilitates the hydrolysis of anandamide, a ligand for cannabinoid receptors. In the gastrointestinal tract, inhibition of FAAH reduces intestinal motility (Capasso et al., 2005) and exhibits anti-inflammatory effects *in vivo* (Massa et al., 2004; D’Argenio et al., 2006). Previous research has indicated that endocannabinoids may inhibit the development of precancerous lesions in mouse colons (Izzo et al., 2008) and reduce the proliferation of colorectal carcinoma cells *in vitro*. In our study, hypomethylated and highly expressed FAAH correlated with a favorable prognosis, echoing findings in pancreatic cancer (Michalski et al., 2008). This contrasts with the findings of other studies where elevated FAAH expression was linked to poor outcomes in various cancers (Fogli et al., 2006; Carracedo et al., 2006).

T cells CD4 memory resting have been found to have lower expression in tumor tissues and high-risk group in gastric cancer specimens. The Pearson correlation analysis showed the negative correlation between this kind of T cells and tumor tissues in the high-risk group. Cancer immunology and immunotherapy are driving forces of research and development in oncology, and previous studies indicated the importance of CD4^+^ T cells, CD4+ T cells play an essential role in the immune system by coordinating both adaptive and innate responses (Künzli and Masopust, 2023). Moreover, they have now been recognized as anti-tumor effector cells in their own right (Speiser et al., 2023). Recent research has shown that CD4^+^ T cells, particularly CD4^+^ memory T cells, are crucial for the immunotherapy-induced tumor regression (Nguyen et al., 2019), which was consistent with the findings of our study.

Our comprehensive analyses of data on DNA methylation arrays, mRNA expression, and related clinical details sourced from the TCGA database have revealed potential predictive biomarkers for early-stage gastric cancer prognosis. These findings could improve treatment accuracy and enhance overall survival in early-stage gastric cancer. However, our study’s limitations include potential selection bias due to its retrospective design and small sample size. There is a scarcity of data to inform treatment choices for this particular group, necessitating further research with a larger cohort. We plan to establish a database for stage I/II gastric cancer at our center to expand our research sample size.

In conclusion, this study has pinpointed key predictive elements that are vital for forecasting the outcomes of early-stage gastric cancer. Central genes like C1orf35 and FAAH have demonstrated both predictive and prognostic significance as biomarkers based on methylation, setting the stage for accurate diagnosis and targeted treatment of gastric cancer.

## Data Availability

The public datasets of TCGA-STAD and GSE84437 for this study can be found in the Cancer Genome Atlas (TCGA, https://portal.gdc.cancer.gov/) and GEO databases. High throughput sequencing data in the study are submitted to the SRA database, accession number: PRJNA1172107.

## References

[B1] Aoyama T. Yoshikawa T. Fujikawa H. Hayashi T. Ogata T. Cho H. (2014). Prognostic factors in stage IB gastric cancer. World J. Gastroenterology 20, 6580–6585. 10.3748/wjg.v20.i21.6580 24914380 PMC4047344

[B2] Atlas N. C. G. (2014). Comprehensive molecular characterization of urothelial bladder carcinoma. Nature 507, 315–322. 10.1038/nature12965 24476821 PMC3962515

[B3] Bausys R. Bausys A. Vysniauskaite I. Maneikis K. Stratilatovas E. Strupas K. (2018). Surgical treatment outcomes of patients with T1-T2 gastric cancer: does the age matter when excellent treatment results are expected? World J. Surg. Oncol. 16, 79. 10.1186/s12957-018-1388-4 29661204 PMC5902993

[B4] Calcagno D. Q. Gigek C. O. Chen E. S. Burbano R. R. Smith M. D. A. C. (2013). DNA and histone methylation in gastric carcinogenesis. World J. Gastroenterology 19, 1182–1192. 10.3748/wjg.v19.i8.1182 23482412 PMC3587474

[B5] Capasso R. Matias I. Lutz B. Borrelli F. Capasso F. Marsicano G. (2005). Fatty acid amide hydrolase controls mouse intestinal motility *in vivo* . Gastroenterology 129, 941–951. 10.1053/j.gastro.2005.06.018 16143133

[B6] Carracedo A. Gironella M. Lorente M. Garcia S. Guzmán M. Velasco G. (2006). Cannabinoids induce apoptosis of pancreatic tumor cells via endoplasmic reticulum stress-related genes. Cancer Res. 66, 6748–6755. 10.1158/0008-5472.CAN-06-0169 16818650

[B7] Cedoz P. L. Prunello M. Brennan K. Gevaert O. (2018). MethylMix 2.0: an R package for identifying DNA methylation genes. Bioinformatics 34, 3044–3046. 10.1093/bioinformatics/bty156 29668835 PMC6129298

[B8] D’Argenio G. Valenti M. Scaglione G. Cosenza V. Sorrentini I. Di Marzo V. (2006). Up-regulation of anandamide levels as an endogenous mechanism and a pharmacological strategy to limit colon inflammation. FASEB J. official Publ. Fed. Am. Soc. Exp. Biol. 20, 568–570. 10.1096/fj.05-4943fje 16403786

[B9] Feinberg A. P. Vogelstein B. (1983). Hypomethylation of ras oncogenes in primary human cancers. Biochem. & Biophysical Res. Commun. 111, 47–54. 10.1016/s0006-291x(83)80115-6 6187346

[B10] Fogli S. Nieri P. Chicca A. Adinolfi B. Mariotti V. Iacopetti P. (2006). Cannabinoid derivatives induce cell death in pancreatic MIA PaCa-2 cells via a receptor-independent mechanism. FEBS Lett. 580, 1733–1739. 10.1016/j.febslet.2006.02.024 16500647

[B11] Gevaert O. (2015). MethylMix: an R package for identifying DNA methylation-driven genes. Bioinformatics 31, 1839–1841. 10.1093/bioinformatics/btv020 25609794 PMC4443673

[B12] Guo J. N. Chen D. Deng S. H. Huang J. R. Song J. X. Li X. Y. (2021). Identification and quantification of immune infiltration landscape on therapy and prognosis in left- and right-sided colon cancer. Cancer Immunol. Immunother. CII 71, 1313–1330. 10.1007/s00262-021-03076-2 34657172 PMC9122887

[B13] Hu X. Y. Ling Z. N. Hong L. L. Yu Q. M. Li P. Ling Z. Q. (2021). Circulating methylated THBS1 DNAs as a novel marker for predicting peritoneal dissemination in gastric cancer. J. Clin. Lab. Anal. 35, e23936. 10.1002/jcla.23936 34390026 PMC8418496

[B14] Huang C. Liu H. Hu Y. Sun Y. Su X. Cao H. (2022). Laparoscopic vs open distal gastrectomy for locally advanced gastric cancer: five-year outcomes from the CLASS-01 randomized clinical trial. JAMA Surg. 157, 9–17. 10.1001/jamasurg.2021.5104 34668963 PMC8529527

[B15] Ikoma N. Blum M. Chiang Y. J. Estrella J. S. Roy-Chowdhuri S. Fournier K. (2016). Survival rates in T1 and T2 gastric cancer: a Western report. J. Surg. Oncol. 114, 602–606. 10.1002/jso.24382 27439746

[B16] Izzo A. A. Aviello G. Petrosino S. Orlando P. Marsicano G. Lutz B. (2008). Increased endocannabinoid levels reduce the development of precancerous lesions in the mouse colon. J. Mol. Med. 86, 89–98. 10.1007/s00109-007-0248-4 17823781 PMC2755791

[B17] Kamarudin A. N. Cox T. Kolamunnage-Dona R. (2017). Time-dependent ROC curve analysis in medical research: current methods and applications. BMC Med. Res. Methodol. 17, 53. 10.1186/s12874-017-0332-6 28388943 PMC5384160

[B18] Künzli M. Masopust D. (2023). CD4+ T cell memory. Nat. Immunol. 24, 903–914. 10.1038/s41590-023-01510-4 37156885 PMC10343737

[B19] Lu Y. Rosenfeld R. Simon I. Nau G. J. Bar-Joseph Z. (2008). A probabilistic generative model for GO enrichment analysis. Nucleic Acids Res. 36, e109. 10.1093/nar/gkn434 18676451 PMC2553574

[B20] Luo S. Q. Xiong D. H. Li J. Li G. Wang Y. Zhang J. M. (2020). C1orf35 contributes to tumorigenesis by activating c-MYC transcription in multiple myeloma. Oncogene 39, 3354–3366. 10.1038/s41388-020-1222-7 32103167

[B21] Manel E. (2002). CpG island hypermethylation and tumor suppressor genes: a booming present, a brighter future. Oncogene 21, 5427–5440. 10.1038/sj.onc.1205600 12154405

[B22] Massa F. Marsicano G. Hermann H. Cannich A. Monory K. Cravatt B. F. (2004). The endogenous cannabinoid system protects against colonic inflammation. J. Clin. investigation 113, 1202–1209. 10.1172/JCI19465 15085199 PMC385396

[B23] Meier T. Timm M. Montani M. Wilkens L. (2021). Gene networks and transcriptional regulators associated with liver cancer development and progression. BMC Med. Genomics 14, 41. 10.1186/s12920-021-00883-5 33541355 PMC7863452

[B24] Melton P. E. Johnson M. P. Gokhale-Agashe D. Rea A. J. Ariff A. Cadby G. (2019). Whole-exome sequencing in multiplex preeclampsia families identifies novel candidate susceptibility genes. J. Hypertens. 37, 997–1011. 10.1097/HJH.0000000000002023 30633125

[B25] Michalski C. W. Oti F. E. Erkan M. Sauliunaite D. Bergmann F. Pacher P. (2008). Cannabinoids in pancreatic cancer: correlation with survival and pain. Int. J. Cancer 122, 742–750. 10.1002/ijc.23114 17943729 PMC2225529

[B26] Miyahara K. Ishida M. Kono Y. Hirata T. Obayashi Y. Gotoda T. (2022). Prognosis after curative resection for stage IA gastric cancer in elderly patients: endoscopic submucosal dissection versus surgery. Surg. Today 52, 1329–1340. 10.1007/s00595-022-02456-0 35089444

[B27] Nakajima T. Kinoshita T. Nashimoto A. Sairenji M. Yamaguchi T. Sakamoto J. (2010). Randomized controlled trial of adjuvant uracil–tegafur versus surgery alone for serosa‐negative, locally advanced gastric cancer. Br. J. Surg. 94, 1468–1476. 10.1002/bjs.5996 17948223

[B28] Newman A. M. Liu C. L. Green M. R. Gentles A. J. Feng W. Xu Y. (2015). Robust enumeration of cell subsets from tissue expression profiles. Nat. Methods 12, 453–457. 10.1038/nmeth.3337 25822800 PMC4739640

[B29] Nguyen Q. P. Deng T. Z. Witherden D. A. Goldrath A. W. (2019). Origins of CD4(+) circulating and tissue-resident memory T-cells. Immunology 157, 3–12. 10.1111/imm.13059 30897205 PMC6459775

[B30] Orman S. Cayci H. M. (2019). Gastric cancer: factors affecting survival. Acta Chir. Belg 119, 24–30. 10.1080/00015458.2018.1453437 29560799

[B31] Qiang Z. Jubber I. Lloyd K. Cumberbatch M. Griffin J. (2023). Gene of the month: GATA3. J. Clin. Pathol. 76, 793–797. 10.1136/jcp-2023-209017 37726118

[B32] Saito H. Murakami Y. Miyatani K. Kuroda H. Matsunaga T. Fukumoto Y. (2016). Predictive factors for recurrence in T2N0 and T3N0 gastric cancer patients. Langenbecks Archives Surg. 401, 823–828. 10.1007/s00423-016-1480-6 27460840

[B33] Sakuramoto S. Sasako M. Yamaguchi T. Kinoshita T. Fujii M. Nashimoto A. (2007). Adjuvant chemotherapy for gastric cancer with S-1, an oral fluoropyrimidine. N. Engl. J. Med. 357, 1810–1820. 10.1056/NEJMoa072252 17978289

[B34] Seager R. J. Hajal C. Spill F. Kamm R. D. Zaman M. H. (2017). Dynamic interplay between tumour, stroma and immune system can drive or prevent tumour progression. Converg. Sci. Phys. Oncol. 3, 034002. 10.1088/2057-1739/aa7e86 30079253 PMC6070160

[B35] Smyth G. K. Ritchie M. Thorne N. Wettenhall J. (2005). “LIMMA: linear models for microarray data,” in Bioinformatics and computational biology solutions using R and bioconductor. Statistics for biology and Health.

[B36] Song D. Zhou Z. Wu J. Wei T. Zhao G. Ren H. (2022). DNA methylation regulators-related molecular patterns and tumor immune landscape in hepatocellular carcinoma. Front. Oncol. 12, 877817. 10.3389/fonc.2022.877817 36091162 PMC9459088

[B37] Speiser D. E. Chijioke O. Schaeuble K. Münz C. (2023). CD4(+) T cells in cancer. Nat. Cancer 4, 317–329. 10.1038/s43018-023-00521-2 36894637

[B38] Steidl C. Lee T. Shah S. P. Farinha P. Han G. Nayar T. (2010). Tumor-associated macrophages and survival in classic Hodgkin's lymphoma. N. Engl. J. Med. 362, 875–885. 10.1056/NEJMoa0905680 20220182 PMC2897174

[B39] Suarez-Alvarez B. Rodriguez R. M. Fraga M. F. López-Larrea C. (2012). DNA methylation: a promising landscape for immune system-related diseases. Trends Genet. 28, 506–514. 10.1016/j.tig.2012.06.005 22824525

[B40] Tahara H. Kay M. A. Yasui W. Tahara E. (2013). MicroRNAs in cancer: the 22nd Hiroshima cancer seminar/the 4th Japanese association for RNA interference joint international symposium, 30 august 2012, grand prince hotel hiroshima. Jpn. J. Clin. Oncol. 43, 579–582. 10.1093/jjco/hyt037 23487440

[B41] Tahara T. Arisawa T. (2015). DNA methylation as a molecular biomarker in gastric cancer. Epigenomics 7, 475–486. 10.2217/epi.15.4 26077432

[B42] Wang G. Zhang W. Zhou B. Jin C. Wang Z. Yang Y. (2015). The diagnosis value of promoter methylation of UCHL1 in the serum for progression of gastric cancer. Biomed. Res. Int. 2015, 741030. 10.1155/2015/741030 26550574 PMC4624918

[B43] Yan H. Chen W. Ge K. Mao X. Li X. Liu W. (2021). Value of plasma methylated SFRP2 in prognosis of gastric cancer. Dig. Dis. Sci. 66, 3854–3861. 10.1007/s10620-020-06710-8 33216241

[B44] Yuan S. Gao Y. Xia Y. Wang Z. Wang X. (2022). DNA methylation regulator-mediated modification pattern defines tumor microenvironment immune infiltration landscape in colon cancer. Front. Genet. 13, 1008644. 10.3389/fgene.2022.1008644 36276973 PMC9582351

[B45] Zhu Y. Qiu P. Ji Y. (2014). TCGA-Assembler: open-source software for retrieving and processing TCGA data. Nat. Methods 11, 599–600. 10.1038/nmeth.2956 24874569 PMC4387197

