# Correction: Development of a prognostic model for early-stage gastric cancer-related DNA methylation-driven genes and analysis of immune landscape

**DOI:** 10.3389/fmolb.2025.1658908

**Published:** 2025-12-09

**Authors:** Chen Su, Zeyang Lin, Zhijian Ye, Jing Liang, Rong Yu, Zheng Wan, Jingjing Hou

**Affiliations:** 1 The School of Clinical Medical, Fujian Medical University, Fuzhou, Fujian, China; 2 Department of Gastrointestinal Surgery, Zhongshan Hospital of Xiamen University, School of Medicine, Xiamen University, Xiamen, China; 3 Institute of Gastrointestinal Oncology, School of Medicine, Xiamen University, Xiamen, China; 4 Department of Pathology, Zhongshan Hospital of Xiamen University, School of Medicine, Xiamen University, Xiamen, China; 5 Department of Pathology, The Third Affiliated Hospital of Sun Yat-sen University, Guangzhou, China; 6 Department of Minimally Invasive and Interventional Therapy for Cancer, Zhongshan Hospital of Xiamen University, School of Medicine, Xiamen, China

**Keywords:** gastric cancer, FAAH, C1orf35, DNA methylation-driven gene, prognosis

In the **Funding** statement, the name of one of the funders was incorrectly provided as Foundation of Xiamen Municipal Bureau of Science and Technology. The correct funder is Natural Science Foundation of Xiamen, China.

An incorrect number was provided for the Natural Science Foundation of Fujian Province. The correct number is No. 2023J011600.

The original article has been updated.

